# Dissecting human adipose tissue heterogeneity using single‐cell omics technologies

**DOI:** 10.1186/s13287-024-03931-w

**Published:** 2024-09-27

**Authors:** Giuliana Di Rocco, Angelo Trivisonno, Giovanni Trivisonno, Gabriele Toietta

**Affiliations:** 1 Unit of Cellular Networks and Molecular Therapeutic Targets, IRCCS Regina Elena National Cancer Institute, 00144 Rome, Italy; 2 Fondazione Policlinico Universitario Agostino Gemelli IRCCS, 00168 Rome, Italy; 3 School of Medicine, University of Rome Campus Biomedico, 00128 Rome, Italy; 4 Tumor Immunology and Immunotherapy Unit, IRCCS Regina Elena National Cancer Institute, Via E. Chianesi, 53, 00144 Rome, Italy

**Keywords:** Adipose tissue, Regenerative medicine, Single-cell sequencing, Tissue heterogeneity, Omics technologies

## Abstract

Single-cell omics technologies that profile genes (genomic and epigenomic) and determine the abundance of mRNA (transcriptomic), protein (proteomic and secretomic), lipids (lipidomic), and extracellular matrix (matrisomic) support the dissection of adipose tissue heterogeneity at unprecedented resolution in a temporally and spatially defined manner. In particular, cell omics technologies may provide innovative biomarkers for the identification of rare specific progenitor cell subpopulations, assess transcriptional and proteomic changes affecting cell proliferation and immunomodulatory potential, and accurately define the lineage hierarchy and differentiation status of progenitor cells. Unraveling adipose tissue complexity may also provide for the precise assessment of a dysfunctional state, which has been associated with cancer, as cancer-associated adipocytes play an important role in shaping the tumor microenvironment supporting tumor progression and metastasis, obesity, metabolic syndrome, and type 2 diabetes mellitus. The information collected by single-cell omics has relevant implications for regenerative medicine because adipose tissue is an accessible source of multipotent cells; alternative cell-free approaches, including the use of adipose tissue stromal cell-conditioned medium, extracellular vesicles, or decellularized extracellular matrix, are clinically valid options. Subcutaneous white adipose tissue, which is generally harvested via liposuction, is highly heterogeneous because of intrinsic biological variability and extrinsic inconsistencies in the harvesting and processing procedures. The current limited understanding of adipose tissue heterogeneity impinges on the definition of quality standards appropriate for clinical translation, which requires consistency and uniformity of the administered product. We review the methods used for dissecting adipose tissue heterogeneity and provide an overview of advances in omics technology that may contribute to the exploration of heterogeneity and dynamics of adipose tissue at the single-cell level.

## Introduction

Over the last decades, the therapeutic potential of autologous adipose tissue-derived material transplantation has been explored in several regenerative medicine clinical trials [1]. Most of the preclinical and translational research employed adipose tissue-derived stromal cells isolated by enzymatic digestion of white adipose tissue (WAT), mainly obtained from subcutaneous fat depots [2, 3]; the regenerative potential of mechanically manipulated adipose tissue has also been evaluated [4]; moreover, adipose tissue paracrine factors, including extracellular vesicles (EVs) and cell-conditioned medium, and decellularized extracellular matrix, have also been considered as cell-free therapeutics [5], in order to circumvent the limited transplanted cell viability in the hypoxic microenvironment at the administration site [6, 7]. Therapeutic efficacy of adipose tissue derivatives in regenerative medicine, dermatology, plastic and aesthetic surgery procedures has been ascribed to both the action of resident multipotent stromal cells and paracrine signals. Nonetheless, inconsistencies in the methods used for adipose tissue manipulation currently limit the reproducible therapeutic efficacy of adipose tissue derivatives, but progressive efforts in the standardization of the procedures will eventually address this issue. Another source of variability is the intrinsic biological heterogeneity, which requires extensive studies at the cellular level to unravel the biological properties, function and clinical potential of adipose tissue derivatives [8]. Single-cell technologies measure the abundance of molecules in individual cells and represent a unique tool to finely dissect tissue cellular and functional heterogeneity, which has been more extensively used for the analysis of the human tumor microenvironment [9]. Single-cell omics analysis of adipose tissue heterogeneity may support the identification and isolation of more potent cellular subpopulations and paracrine factors with preferred regenerative properties [10–12]. Therefore, the exploitation of technologies for single-cell analysis can be instrumental in more precisely elucidating the mechanisms underlying stem cell differentiation potentials, possibly finding druggable targets to promote tissue regeneration [13] and clinical translation [14].

Here, we review the current methods and provide an overview of the advances in omics technology used to better define and refine the regenerative potential of adipose tissue derivatives.

## Intrinsic and extrinsic factors affecting adipose tissue derivative heterogeneity

The comprehensive evaluation of adipose tissue is challenging because is a heterogeneous and dynamic organ. Adipose tissue is the main reservoir of energy substrates and endocrine mediators, including growth factors, hormones, cytokines, and microRNAs, which contribute to whole-body energy metabolism and homeostasis. Moreover, adipose tissue has a key role in immune response, mechanical protection, thermogenesis, and thermal insulation. This variety of functions is reflected in its heterogeneous composition: mature adipocytes are the distinctive cell type and encompass up to 80% of the volume of a white adipose tissue depot; however, multiple additional cytotypes are also present, including preadipocytes, endothelial cells, pericytes, immune cells such as T cells and macrophages, and mesenchymal/stromal progenitor/stem cells, which collectively form the adipose tissue stromal vascular fraction (SVF) [15]. Interestingly, although SVF occupies only a minor part of the volume of an adipose tissue depot, it comprises approximately 60–80% of the total cell number. In addition, an extracellular matrix (ECM) network, mainly composed of collagens and proteoglycans, comprises the adipose tissue niche, which exerts biomechanical functions and serves as a reservoir of cytokines, growth factors, and extracellular vesicles that influence cell shape and metabolism. Cross-talk between the different components regulates both adipocyte fate and function and supports tissue dynamic plasticity to respond to the energy needs of the organism through adipocyte hypertrophy, proliferation of precursor cells, and ECM remodeling.

**Fig. 1:**
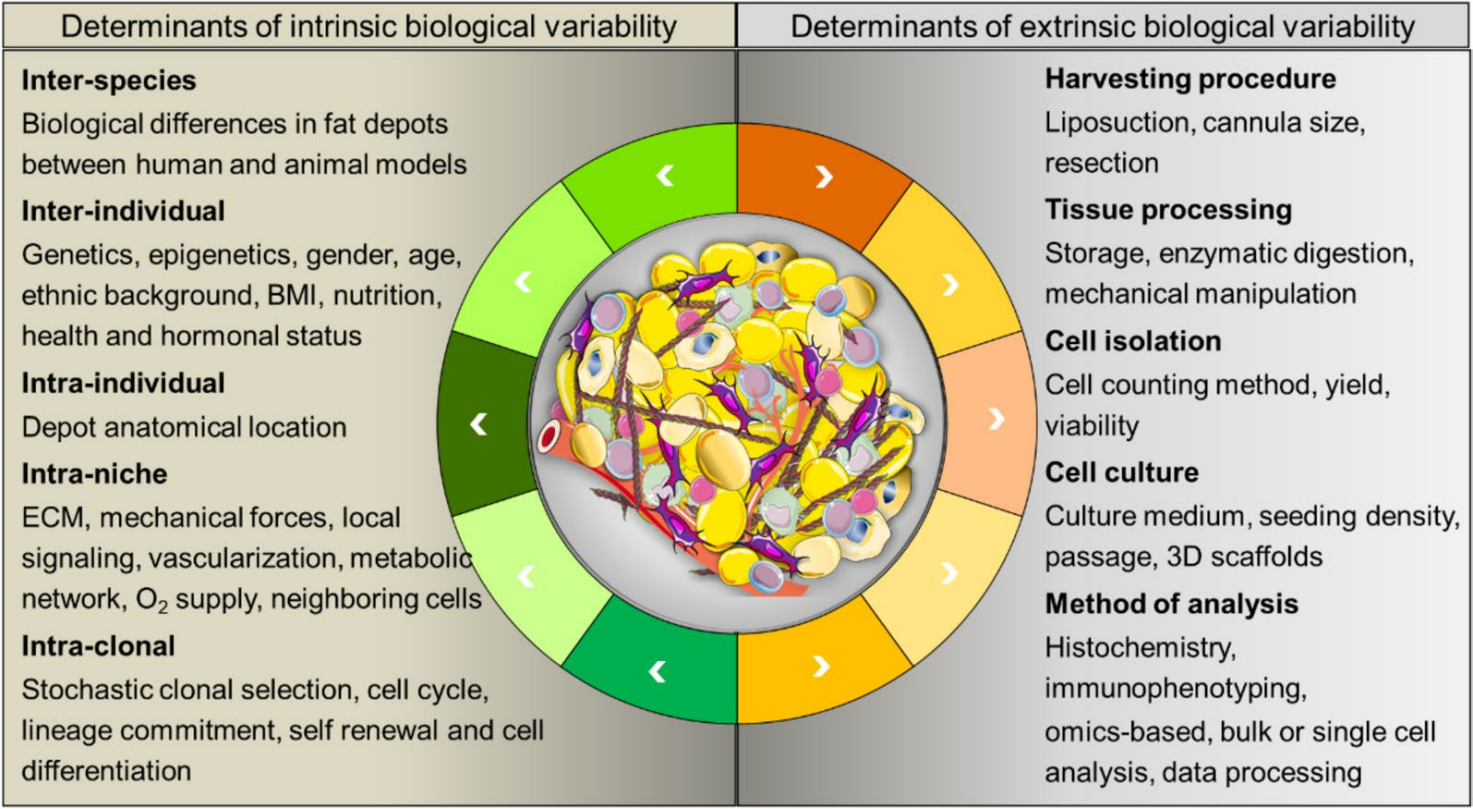
Major determinants affecting adipose tissue heterogeneity. BMI: body mass index; ECM: extracellular matrix. Templates were adapted from Servier Medical Art (https://smart.servier.com/, accessed: 05 September 2024), available under a Creative Commons Attribution 3.0 Unported License

The adipose tissue composition of humans cannot be predicted from studies on mice [16] and other vertebrates [17, 18] due to significant differences in their metabolism and physiology. In humans, adipose tissue depots are highly dynamic and variable in distribution and dimension, depending on several factors, including donor genetics, epigenetics, gender, age, ethnic background, metabolism and nutritional status, and response to hormones or drugs. As a result, there is a substantial donor-to-donor variation in adipose tissue composition; moreover, different cell populations are present in different fat depots and local cellular composition variety exists within the same white adipose tissue depot, resulting in intraindividual variation; furthermore, cell populations respond to their specific tissue microenvironment and neighboring cells. Therefore, it is possible that adipose tissue heterogenicity may arise, at least in part, by cell transition in different states in response to intra- and inter-tissue cellular crosstalk (Fig. 1). Health status strongly affects adipose tissue endocrine and paracrine functions, as dysfunctional adipose tissue has been associated with obesity, metabolic syndrome, type 2 diabetes mellitus and cancer-associated cachexia [19, 20]. Active cross talk between adipose tissue and cancer exists, as some tumors (including, prostate, breast, and ovary) invade the surrounding adipose tissue, whereas adipose tissue can promote carcinogenesis via paracrine and metabolic modulation [21]. Furthermore, the stromal component of the tumor microenvironment is characterized by the presence of cancer-associated adipocytes, which play an active role in supporting tumor progression and metastasis, and therapy resistance via metabolic reprogramming and paracrine-mediated crosstalk with tumor cells [22, 23]. Cancer-associated adipocytes are characterized by an activated phenotype, morphological and functional changes, reduced lipid content and expression of adipocyte markers [24]; specific classes of tumor‑promoting adipocytes characterized by expression of dipeptidyl peptidase-4 (DPP4) and adiponectin (ADIPOQ) have been recently identified [25]. In addition, adipose stromal cells shape the tumor immune microenvironment originating collagen 11A-expressing cancer-associated fibroblasts that promote tumor ECM remodeling and modulate antitumor immune response [26]. The contribution of adipocytes in cancer has often been underestimated, as well as their potential role as therapeutic targets [27]; however, recently, Sato defined as "adipo-oncology" a new area of research to improve our understanding of the role of adipocytes to increase the effectiveness of metastatic tumor therapy [23].

Based on tissue anatomical location, white adipose tissue (WAT) is subcategorized into subcutaneous (sWAT or SAT) and visceral or omental (vWAT or VAT) fat; differences in the anatomy and function between sWAT and vWAT [28] distinctively affect the corresponding cell gene and protein expression profiles [29–32]. In humans, distinct subcutaneous and dermal adipose tissue depots are located beneath the skin, mainly in the abdominal, femoral and gluteal regions. Subcutaneous WAT can be easily collected by liposuction, digested by collagenase, followed by washing, filtration, and centrifugation steps to collect the cellular SVF (cSVF) fraction encompassing multipotent cells able to differentiate into adipogenic, chondrogenic, and osteogenic cells [33]. Freshly isolated cSVF can be used either for therapeutic purposes or for in vitro expansion. Adipose tissue-derived stromal cells (ASCs) are isolated by culturing cSVF and maintaining only plastic-adherent cells. The yield of ASCs depends on several parameters, including the harvesting procedure, isolation protocol, and cell counting method [34, 35]. Moreover, the conditions used for ex vivo cell culture amplification influence various aspects of ASC gene expression, functional, proliferative and differentiative potentials [36, 37]. Alternatively, the adipose tissue stromal vascular fraction (tSVF) can be obtained by minimal, nonenzymatic manipulation of lipoaspirate [4]. This procedure eliminates the prevalent adipocytic fraction and preserves, in addition to the SVF cellular components, the ECM, which has biomechanical functions and can store soluble factors with immune regulatory, angiogenic and tissue reparative capabilities. Non-enzymatic manipulation can be performed using different devices and protocols; however, standardization of the procedures is needed for a reliable prediction of the treatment outcome [38].

## Methods for dissecting adipose tissue heterogeneity

Several methods have been used to study adipose tissue morphological, cellular and functional heterogeneity (Table 1). Some technologies, mainly based on histological examination, can be performed on adipose tissue samples and allow for the analysis of tissue morphology and three-dimensional (3D) structure. Other applications analyze mixtures of intact cells, either freshly isolated or upon ex vivo culture, after adipose tissue disaggregation. Innovative transcriptomic techniques aim to provide a more comprehensive understanding of the contribution of each cell type by single-cell analysis [12, 39]. In the following sections, we briefly review the main methods used for dissecting adipose tissue heterogeneity.

**Table 1:** Approaches for the study of adipose tissue heterogeneity

Method	Analysis and type of sample	Main information
Histology and immunohistochemistry	Microscopic analysis of tissue sections	Morphology, staining of structures and cell-specific marker expression
Whole mount imaging	Confocal microscopy analysis of fluorescently-labelled tissue specimens	3D ultrastructure
Functional and differentiation assays	Colony-forming unit-fibroblastoid and trilineage differentiation assays performed on cultured cells	Proliferation and differentiation potential of ASCs
Phenotypical characterization	Cytofluorimetric analysis on dissociated cells	Expression of selected markers. Possibility of cell sorting
Omic-based	Analysis of mRNA, protein, lipid and decellurized ECM from bulk samples or single cells	Unbiased data on transcriptome, proteome/secretome, lipidome and matrisome. Possibility to collect spatially-resolved information

ASC adipose tissue-derived stromal cells; ECM extracellular matrix

## Adipose tissue histology

Earlier attempts to characterize white adipose tissue cellular components have mostly relied on histology to study the morphology of the cells, their anatomical localization [40] and quantify adipocyte cellularity [41]. Subcutaneous white adipose tissue, which represents over 80% of the total fat, is mainly organized into mature unilocular adipocytes and connective tissue septa. In unilocular adipocytes, a large lipid-filled vacuole occupies most of the cytoplasm and the nucleus is localized in the periphery. Under light microscope examination of routine hematoxylin and eosin-stained adipose tissue sections, adipocytes appear as ‘empty cells’ since their typical lipid droplets are extracted by the organic solvents used for tissue processing. The collagen fibers in the septa of loose connective tissue that separate the adipose tissue lobules can be visualized by Picrosirius red or Masson’s trichrome staining. Paraffin-embedded sections of adipose tissue can also be used for immunofluorescence and immunohistochemistry-based approaches to detect the expression of specific adipose tissue markers, including CCAAT enhancer binding protein beta (C/EBP β), peroxisome proliferator-activated receptor-gamma (PPAR-γ), caveolin-1, perilipin-1 and preadipocyte factor-1 (Pref-1) to label progenitor and mature adipocytes [42]. Nonetheless, frozen sectioning generally allows for more effective antigen labeling with fluorochrome-conjugated antibodies compared with formalin-included sections.

Electron microscopy (transmission and scanning) techniques achieve considerably higher resolution than optical histologic analysis. Using electron microscopy, Sbarbati et al. characterized subcutaneous WAT from several depots, highlighting specific 3D morphological and ultrastructural differences mainly related to the composition of the stromal and microvascular compartments of adipose tissue [43]. Accordingly, sWAT harvested from areas including trochanters, inner faces of the knees, thighs, and hips, in virtue of their strong vascular and stromal fraction, may represent the optimal source of adipose tissue to be used for regenerative procedures.

## Whole mounted adipose tissue imaging

Adipose tissue specimens stained with fluorophores can be visualized by whole mount imaging using confocal microscopy [44]. The processing of adipose tissue for whole mounted imaging does not require fixation, embedding, or sectioning; thus, adipose tissue morphology is preserved, allowing for 3D visualization of tissue architecture and providing indications on possible interactions between different cellular components. Furthermore, organotypic slice cultures of human adipose tissue represent a suitable model in which tissue morphology and metabolism are preserved for up to 2 weeks in culture [45]. The major obstacle is that fluorescently conjugated antibodies or fluorescent stains do not fully permeate the tissue. Notwithstanding this limitation, whole mount imaging has been instrumental for the identification of the adipocyte cellular lineage in vivo in murine models [46].

## Adipose tissue stromal cell functional and differentiation assays

The study of adipose tissue-derived cells obtained by enzymatic dissociation may provide more in-depth insights into the contribution of different cell types to adipose tissue function. Adipose tissue-derived stromal cells (ASCs) have a strong potential impact on regenerative cell therapy. These cells are plastic-adherent when maintained in standard culture conditions and should be characterized by functional assays to assess self-renewal and differentiation potentials before clinical use [47].

### Colony-forming unit-fibroblastoid assay

The colony-forming unit-fibroblastoid (CFU-F) assay assesses ASC self-renewal potential by defining the number of progenitor cells. In this assay, ASCs are seeded at low density (40–400 cells/cm^2^), cultured for 10–14 days, and their clonal expansion is determined by counting the colonies after fixation and staining with crystal violet [47]. The self-renewal potential of ASCs is affected by donor age, body mass index and fat localization; moreover, ASCs at higher passage of culture tend to be senescent and show decreased proliferation rate.

### Differentiation assays

In the presence of well-defined culture conditions in vitro, isolated ASCs undergo mesenchymal tri-lineage (adipocytes, osteoblasts, and chondrocytes) differentiation as well as into various nonmesodermal lineages (including cardiomyocytes, myocytes, endothelial cells, neurons, insulin producing cells, and hepatocytes) [33, 48–52].

According to the International Federation for Adipose Therapeutics and Science (IFATS) and the International Society for Cellular Therapy (ISCT) guidelines for proper ASC characterization, adipocytic, chondrogenic and osteoblastic differentiation assays should be performed [47]. Accordingly, cells are maintained in culture in the appropriate inducing medium for approximately 2–4 weeks, and differentiation is then demonstrated by specific staining or by evaluating the expression of distinctive gene markers by reverse transcription quantitative polymerase chain reaction (RT-qPCR) (Table 2). The differentiation potential may be affected by cell culture passage and confluency. Slightly dissimilar differentiation protocols have been published, and some selective media and kits for confirmatory assays are commercially available [33, 53].

**Table 2:** Adipose tissue-derived stromal cells mesenchymal tri-lineage differentiation

Lineage differentiation	Main components of inducing medium	Confirmatory assays	Main markers
Adipogenic	Dexamethasone, 3-isobutyl-1-methylxanthine, insulin, indomethacin, rosiglitazone	Oil red O, Nile red, BODIPY	Adiponectin, C/EBP α, FABP4, leptin, PPAR γ
Chondrogenic	Dexamethasone, L-ascorbic acid 2-phosphate, members of the TGF-β family	Alcian blue, Toluidine blue, Safranin O	Aggrecan, collagen type II, Sox 9, decorin, byglycan
Osteogenic	Dexamethasone, ascorbic acid 2-phosphate, beta-glycerophosphate, 1,25 dihydroxy vitamin D3	Alizarin red, von Kossa	Alkaline phosphatase, sialoprotein, osteocalcin, osterix, runx2

C/EBP α: CCAAT enhancer binding protein alpha; FABP4: fatty acid binding protein 4*;* PPAR γ: peroxisome proliferator activated receptor gamma; *TGF-β:* transforming growth factor beta

The adipogenic induction medium typically contains glucocorticoids, 1-methyl-3-isobutylxanthine and insulin; rosiglitazone or other thiazolidinediones can be included as peroxisome proliferator-activated receptor-gamma (PPAR-γ) agonists. After approximately 2–3 weeks of culture, the formation of lipid droplets can be observed by phase-contrast microscopy after staining with Oil Red O or Nile red. Semi-quantification of the stained lipid droplets can be performed by measuring the absorbance of the eluted Oil red O stain. Recently, a deep learning-based method was developed to more accurately assess the kinetics of adipogenic differentiation of human ASCs [54]. Expression assessment of adipogenic transcription factors (PPAR γ, C/EBP α/β) and adipocyte genes (adiponectin, fatty acid binding protein 4, lipoprotein lipase, leptin, perilipin, adipose triglyceride lipase) can further validate adipogenic differentiation [55].

Different protocols for the chondrogenic differentiation of ASCs have been described [56]; the most common inducing medium comprises dexamethasone, ascorbate-2-phosphate, transforming growth factor beta 1 (TGF-β1), and insulin [57]. After 3–4 weeks of culture, the production of cartilage-like, sulfated proteoglycan-rich matrix can be detected using alcian blue, toluidine blue, or safranin-O staining. The presence of collagen (manly type II and X) can be assessed using Picrosirius red staining. The differentiation of ASCs to chondrocytes can be determined by the expression analysis of collagen type II and X, aggrecan, Sox-9, decorin, and biglycan.

Osteogenic induction medium comprises dexamethasone, ascorbate, beta-glycerophosphate, and 1,25 dihydroxy vitamin D3 [58]. After 2–4 weeks of culture, differentiated ASCs deposit calcium phosphate in the extracellular matrix, which can be detected by Alizarin red or von Kossa staining. The expression of osteogenic markers, including alkaline phosphatase, bone morphogenic proteins, and their receptors, can be assessed.

## Phenotypic characterization of adipose tissue cells by flow cytometry

Collagenase digestion of adipose tissue permits the separation of adipocytes from the cellular stromal vascular fraction, although the isolation of lipid-filled adipocytes is technically difficult because of their fragility and high buoyancy. Isolated cells can then be characterized by flow cytometry (fluorescence-activated cell sorting—FACS), which allows the assessment of cell size and granularity, and the detection of multiple putative candidate lineage markers via fluorescent antibody staining. Limitations of flow cytometry techniques include the fact that lineage markers are mainly cell surface antigens, are not highly specific, and have heterogeneous expression. Thus, multiple markers, which can differ across studies, may be needed to detect specific cell populations; moreover, many cell surface proteins remain undetectable due to the limited commercial availability of specific antibodies. Nonetheless, FACS evaluation has critically contributed to the definition of the stromal cells isolated from adipose tissue that, according to the IFATS and ISCT guidelines, should express stromal cell surface markers (CD13, CD29, CD44, CD73, CD90, CD105), and should not express hematopoietic and endothelial antigens (CD31, CD45, CD235a) [47]. However, it should be noted that even ASCs that meet these criteria are heterogeneous, as variations in the phenotype might be observed at the origin or acquired during cell culture [59]. Accordingly, phenotypic characterization by FACS analysis should be performed to confirm ASC identity at different passages during in vitro expansion for the manufacture of cell-based therapies.

Purification/enrichment of specific ASC subpopulations based on their phenotype can be performed using immunoaffinity methods that rely on the binding of surface markers by specific monoclonal antibodies linked to fluorescent dyes or magnetic microbeads for fluorescent- or magnetic–activated cell sorting, FACS or MACS, respectively [60, 61]. It is also possible to perform negative selection of cells expressing ASC markers to isolate and characterize adipocytes even if fragility and large size make their purification challenging [62], or immune populations [63] within adipose tissue. Recently, the development of a microfluidic device for high-throughput, label-free enrichment of ASCs has been described [64].

## Adipose tissue transcriptomics

The suffix “omic” defines new branches of research aiming at the identification and quantification of biomolecules that contribute to the molecular complexity and heterogeneity of biological samples. Single-cell and spatial omics technologies, in single-modality or in combination (multi-omics approach) [9, 65], as well as the ability of computational tools for data analysis and integration are rapidly evolving. These cutting edge techniques including genomic and epigenomic for the assessment of the profile of genes, proteomic and secretomic to detect the abundance of proteins, lipidomic to identify lipids, matrisomic for detection of extracellular matrix, metabolomics for metabolites, and transcriptomic for analysis of mRNAs, offer the unique opportunity to in-depth characterization of adipose tissue samples under physiological or pathological condition [10].

Currently, it is not possible to identify ASCs using a single marker. The progressive development of microarrays that enabled genome-scale expression analysis, improved high-throughput sequencing capabilities, and, more recently, single-cell RNA sequencing technologies provide an unprecedented opportunity to more accurately elucidate the phenotype, status and function of adipose tissue cellular components by simultaneous assessment of thousands of marker genes at as low as the cellular level. To collect the cellular transcriptomic signature, RNA is extracted and mRNA is selectively reverse-transcribed to generate a cDNA library that can be subsequently sequenced using high-throughput platforms. Then, sequencing data are mapped, the expression level of each gene is determined, and comparative transcriptome evaluation is performed. This analysis allows the identification of known and novel cell populations defining their unique transcriptomic profiles by unsupervised cell clustering. Moreover, using bioinformatics tools it is possible to gain a more comprehensive view of gene expression through the classification and organization into physiologically or pathologically significant networks. Bulk RNA transcriptome data average gene expression across all the cells within a tissue sample; thus, cellular heterogeneity cannot be precisely defined. Both bulk and single-cell RNA-sequencing require tissue disaggregation for efficient RNA extraction; thus, critical spatial information about adipose tissue is lost in the process. Spatial transcriptomics, which combines transcriptional analysis and in situ hybridization, has been developed to provide whole expression data with spatial information, ideally at a single-cell resolution. The transcriptome is highly dynamic because cells may respond to various environmental and pathological stimuli by modulating gene expression. Therefore, transcriptomic analysis may capture adipose tissue dysfunctional biological pathways associated with pathological states such as obesity and diabetes. In the following section, we review how the exploitation of innovative transcriptomics techniques has expanded our knowledge of adipose tissue.

### Bulk RNA transcriptome analysis of adipose tissue and adipose tissue-derived stromal cells

Bulk RNA transcriptomics enables gene expression analysis in adipose tissue samples or pooled cell populations. In this approach, the entire biological sample is processed and RNA extracted to study gene expression using whole genome expression microarrays or RNA-sequencing. Thus, the results reveal the presence of specific RNAs and define the average spectrum of expression of gene markers across all the cells in the sample, yet the contribution of rare cell populations can be unnoticed. Comparison of bulk transcriptomic profiles is informative for assessing global variability among fat depots [66, 67] and mesenchymal stromal cells of different origin [68]; for instance, a higher aggregated profile of expression of genes important in endocrine function has been assessed in visceral compared with subcutaneous adipose tissue, in agreement with known functional differences between the two depots [69]. Bulk RNA transcriptomics can also be used to define gene expression dynamics during ASC isolation, expansion and differentiation. In particular, four distinct adipocyte subtypes have been identified by bulk transcriptomic analysis of human ASC clones before and after adipogenic differentiation, thus confirming adipocyte heterogeneity, which can be traced to specific progenitor cells [70].

Bulk transcriptomics cannot distinguish expression profiles in unique cell populations; nevertheless, computational approaches of deconvolution, which use expression datasets from known cell types as reference, allow for an estimate of cell type-specific gene expression from bulk RNA-seq data [71]. Performing deconvolution assessment of bulk human SAT transcriptome datasets, Glastonbury et al. characterized subcutaneous adipose tissue heterogeneity by estimating the proportions of adipocytes, macrophages, CD4^+^ T cells, and microvascular endothelial cells [72]. Moreover, a bulk RNA-seq deconvoluting algorithm that relies on single-nucleus RNA sequencing (snRNA-seq) data has been recently developed to estimate the composition of over 13 distinct cell types in human SAT and VAT [73]. Furthermore, by gene expression deconvolution of publicly available bulk transcriptomics data, Lenz et al. [74] estimated that the median fraction of adipocytes in different adipose tissue depots is within the range of 55–80%. Interestingly, the average percentage of ASCs is approximately 15% in SAT, making adipose tissue the richest source of multipotent stromal cells in the body, even if a large variability (from 3% to over 30%) has been observed across individuals. Recently, on the bases of deconvoluted bulk RNA-seq data, Li et al*.* compared the SVF cellular composition between Coleman fat and ECM/SVF gel obtained by mechanical processing*,* showing that the proportion of the stromal cells was not modified by the procedure, while a significant loss of M2 macrophages was observed in the ECM/SVF-gel [75].

**Fig. 2:**
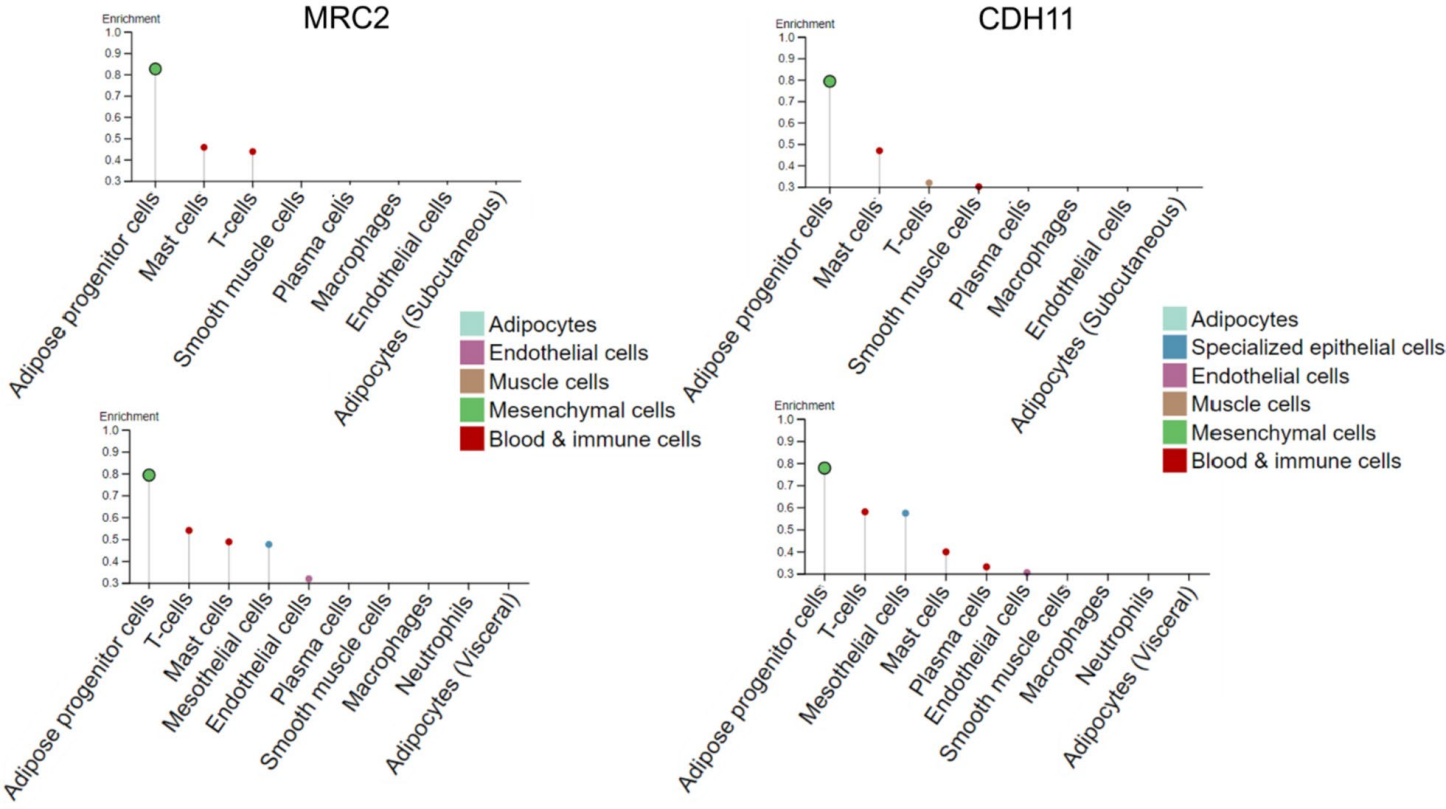
Adipose tissue cell type enriched transcriptome**.** Genes with predicted adipose progenitor cell specificity within subcutaneous (top panels) or visceral adipose tissue (bottom panels). MRC2: mannose receptor C type 2; CDH11: cadherin 11; (image credit: courtesy of Human Protein Atlas, www.proteinatlas.org, accessed: 05 September 2024 [136]. Image available at the following URL: v23.proteinatlas.org/humancell [76, 137]

Integrative co-expression analysis of publicly available adipose tissue bulk RNA-seq data was used to implementing a human adipose tissue cell-type transcriptome atlas and define the expression profile of genes differentially expressed between SAT and VAT [76]. Collectively, these studies highlighted depot-specific gene expression profiles and were instrumental in the identification of putative innovative markers suitable for detecting distinct cell populations. In particular, adipose progenitor cells have been shown to specifically express higher levels of mannose receptor C type 2 (MRC2, aliases ENDO180, UPARAP, CD280), which plays a role in extracellular matrix remodeling, and cadherin 11 (CDH11), which is involved in calcium-dependent cell–cell adhesion (Fig. 2). Similarly, the immunoglobulin superfamily member CD26 (alias B7 Homolog 3, B7-H3) is one of the 11 genes with the highest predicted specificity for mesenchymal cells within VAT [76].

### Single-cell RNA-sequencing and single-nucleus RNA sequencing of adipose tissue cells

Over the past few years, the progression of molecular biology techniques with improved resolution, such as single-cell real-time quantitative PCR (RT-qPCR), digital droplet PCR (ddPCR) and, particularly, single-cell RNA sequencing (scRNA-seq), has provided the possibility of dissecting adipose tissue heterogeneity through the transcriptional evaluation of individual cells. While PCR-based techniques are limited to the study of a relatively small number of genes, high-throughput scRNA-seq offers a holistic view of the adipose tissue transcriptome, capturing the entire spectrum of cell states and enabling the identification of novel markers and cellular populations. In virtue of its sensitivity, scRNA-seq can also investigate low-copy mRNAs. Single-cell suspensions are needed for scRNA-seq analysis; thus, after extensive adipose tissue disaggregation or cultured cell trypsinization, each cell is isolated by different methods, including fluorescence-activated cell sorting, magnetic-activated cell sorting, and microfluidic systems, and the RNA is amplified and sequenced from each cell separately.

Single-cell transcriptome investigation provides a snapshot of the gene expression profile of an individual cell; nonetheless, by sequencing several cells at a given time point, it is possible to infer the transcriptome dynamics of biological processes such as cell differentiation, development, and disease progression. In particular, assuming a continuous spectrum of transcriptional changes between more or less differentiated conditions during cell differentiation, computational evaluation of RNA-seq data makes it possible to associate distinctive cells, characterized by their representative transcriptome, with different states in a pseudo-temporal trajectory of cell differentiation, generating a presumed cell lineage tree. This approach has been successfully used to establish a lineage hierarchy of several distinct subtypes of ASC [77]. Conversely, it is difficult to define the precise trajectory of adipogenesis because adipocytes have a slow turnover rate [78] and a single sample cannot always comprise the complete transcriptional changes associated with all the differentiation steps [79].

To obtain a comprehensive view of adipose tissue cellular and temporal heterogeneity, a relatively large number of individual cells must be isolated, processed and the RNA sequenced. Single-cell isolation of adipocytes for scRNA-seq studies is challenging because of their buoyancy and fragility, which are attributable to their high lipid content and large size. To circumvent this problem, in vitro differentiation studies on preadipocytes have been performed [80], but this approach cannot precisely recapitulate all physiological differentiation cues. Therefore, although scRNA-seq approaches have been extensively used to study the adipose tissue stromal-vascular cell fraction, scRNA-seq data for adipocytes are limited.

Single-nucleus RNA sequencing (snRNA-seq), which works on nuclei rather than intact cells, is an alternative approach to scRNA-seq. The snRNA-seq procedure does not require tissue enzymatic digestion, which can lead to critical loss of some cell types; therefore, it is suitable for the analysis of the transcriptome of all cell populations within an adipose tissue sample, including fragile adipocytes and adipogenic precursors [81]. Moreover, nuclei can be isolated from both fresh and fixed or frozen archived tissues. Although scRNA-seq enables the analysis of both nuclear and cytoplasmic transcripts, whereas snRNA-seq mostly measures nuclear transcripts, both approaches yield similar expression profiles of relevant genes in adipose tissue samples [82, 83].

Several studies, preferentially using scRNA-seq for characterizing human stromal vascular fraction cells, and snRNA-seq to characterize mature adipocytes, have been performed, as reported in Table 3. We mainly focused on studies performed on human-derived samples because, due to substantial intra-species differences in adipose tissue structure, function and metabolism [16], cell populations identified in murine models do not exactly map those in humans [25]. Nonetheless, some cell populations identified by scRNA-seq in mice were confirmed in humans; for instance, the detection of adipogenesis-regulatory cells refractory to adipogenesis, named Areg, using scRNA-seq on murine sWAT led to the identification of a functionally conserved CD142^+^, SCA1^+^ cell population in humans [84]; the dipeptidyl peptidase-4 expressing (DPP4^+^) mesenchymal cell population, which in virtue of its highly proliferative potential may be used for regenerative medicine applications, was identified in both murine and human adipose tissue [77].

**Table 3:** Transcriptomics studies of human white adipose tissue

Samples	Transcriptomics	Main identified subpopulations	Refs
SVF from sWAT	scRNA-seq	1 ASCs and 3 resident macrophage subtypes	[88]
ASCs from sWAT	scRNA-seq	> 96% of the cells expressed the ISCT-proposed positive marker genes	[85]
ASCs from sWAT	Bulk RNA-seq	4 adipocyte subtypes and corresponding progenitors	[70]
Freshly sorted ASC from vWAT and sWAT	Bulk RNA-seq	3 adipocyte progenitor cell subtypes	[135]
Commercial sWAT preadipocytes	scRNA-seq	2 classes of subcutaneous adipocytes	[80]
12 vWAT and 13 sWAT from obese donors	scRNA-seq	17 clusters cell clusters, including 7 progenitors	[89]
sWAT and vWAT	snRNA-seq; scRNA-seq for ASC	1 PRDM16-expressing EC progenitor cell population	[90]
WAT	ST	3 fat cell types with specific localization	[99]
FACS sorted SVF from lean and obese WAT	scRNA-seq	28 distinct cell types	[91]
sWAT	scRNA-seq and snRNA-seq	7 distinct adipocyte clusters	[94]
ASCs from sWAT	scRNA-seq	9 cell clusters; maximum stemness in a cluster highly expressing TPM1 and CCND1	[87]
sWAT and vWAT	Bulk RNA-seq	10 major constituent cell types	[76]
sWAT	scRNA-seq and snRNA-seq	3 distinct pre-adipocyte clusters	[82]
ASCs from sWAT and vWAT undergoing in vitro differentiation	scRNA-seq	2 main cell clusters, one adipogenic and one structural branch, developing from a common progenitor	[86]
Leg sWAT from a chronic wound patient	scRNA-seq	Adipocyte precursors expressing CXCL3	[93]
sWAT, vWAT, pvWAT	scRNA-seq, snRNA-seq, ST	60 cell clusters	[96]
sWAT and vWAT from lean and obese subjects	scRNA-seq, snRNA-seq	4 adipocyte populations; increased VECs and FAPs in sWAT	[97]

Abbreviations: ASC: adipose tissue-derived stromal cells (cultured); CCND1: cyclin D1; EC: endothelial cells; FAPs: fibroblasts and adipogenic progenitor cells; ISCT: International Society for Cellular Therapy; PRDM16: PR domain zinc finger protein 16; sWAT/SAT: subcutaneous (white) adipose tissue; vWAT/VAT: visceral (white) adipose tissue; SVF: stromal vascular cells (uncultured); ST: spatial transcriptomics; TPM1: tropomyosin 1; VEC: vascular endothelial cells

Some scRNA-seq studies have been performed on cultured cells, whereas others on freshly isolated uncultured cells. Large-scale scRNA-seq of cultured human ASCs from three donors demonstrated that over 96% of cells adhere to the IFATS and ISCT recommendations for ASC marker expression. Nonetheless, even in an almost pure cell population, gene expression levels may still be heterogeneous, mainly because of cell cycle influence [85]. In addition, in primary, FACS-sorted, human preadipocytes acquired from a commercial source and subjected to adipogenic culture conditions, at least two distinct cell populations can be identified by scRNA-seq analysis [80]. Palani et al*.* recently isolated human adipocyte progenitors from subcutaneous and visceral white adipose tissue (WAT), which were then assessed by scRNA-seq at different timepoints during in vitro differentiation. The authors delineated human adipocyte single-cell trajectory (available at https://cphbat.shinyapps.io/adipodiff/. Accessed: 05 September 2024) showing that when cultured in adipogenic differentiating medium, WAT cells separate from a common progenitor into two main cell fates, an adipogenic and a structural branch, defined on the basis of an extracellular matrix and developmental gene signature [86].

Single-cell transcriptomic sequencing assessment of ASCs isolated from one healthy donor performed after 3 passages of culture revealed 9 cell clusters; moreover, the authors, using a computational approach, demonstrated that the ASC cluster with the maximum stemness was characterized by the highest expression of genes involved in cytoskeleton structure and regulation of cell cycle transition, such as tropomyosin 1 (TPM1) and cyclin D1 (CCND1) [87].

The first scRNA-seq study performed on uncultured cells from human sWAT of 4 healthy women suggested that ASCs constitute a single and homogeneous population [88]. In 2020, Vijay et al*.* used single-cell RNA-seq to analyze the non-adipocyte fraction of adipose samples from 14 obese donors. This study assessed that in the stromal vascular fraction of human WAT, the average proportion of adipocyte progenitors and stem cells is approximately 55%, divided into several subpopulations with different commitment but all CD45^−^, CD34^+^, CD31^−^ [89]. In particular, within sWAT, pre-adipocyte/adipose stem cells express matrix Gla protein (MGP), apolipoprotein D (APOD), C-X-C Motif Chemokine Ligand 14 (CXCL14), WNT1-inducible signaling pathway protein 2 (WISP2, alias CCN5) and complement factor D (CDF), whereas the cluster of more mature adipocyte progenitor cells express apolipoprotein E (APOE), fatty acid binding protein 4 (FABP4), C/EBP β, CD36 antigen (collagen type I receptor, thrombospondin receptor) and share CDF expression; an additional stromal cell cluster, mainly encompassing fibroblasts, is characterized by ECM remodeling and fibrosis markers such as diverse types of collagens (COL3A1, COL6A3, COL1A1 and COL6A1). The study also highlighted the presence of 37% immune cells and 8% endothelial cells, including microvascular and lymphatic endothelial cells [89]. In line with these results, high PR domain zinc finger protein 16 (PRDM16) expression has been identified as a possible marker for an endothelial progenitor cell population that is enriched in sWAT compared with vWAT, in agreement with an increased sWAT angiogenic potential [90].

Hildreth et al*.* performed scRNA-seq analysis on CD45^+^-sorted uncultured SVF isolated from abdominal sWAT from lean and obese patients, thereby identifying 28 distinct structural and immune cell populations [91]. In particular, according to this study, WAT contains a wide array of immune cells, including neutrophils, monocytes, macrophages, different classes of T cells (CD4 + T cells, naive CD8 + T cells, cytotoxic CD8 + T cells, CD8 + γδ T cells, regulatory T cells, mucosa-associated invariant T cells), B lymphocytes, mature NK cells, myeloid-like cells, and NK-like cells. Interestingly, while lean WAT contains mainly noninflammatory cells, in obese WAT the immune profile shifts to a proinflammatory state [92]. Recently, Gu et al*.* analyzed the cell subpopulations in WAT of different anatomical origins using scRNA-seq data from public databases and one de novo dataset from subcutaneous leg adipose tissues and found that sWAT of the legs is rich in ASCs [93]. According to single-cell RNA seq analysis of SVF cells isolated from lipoaspirate obtained from 2 healthy donors, Whytock et al*.* estimated that one stem cell cluster (PTPRC^−^/PECAM1^−^/CD34^+^/PDGFRA^+^/PDGFRB^+^) represents approximately 28% of the total SVF and 7% of the total sWAT cells [82].

**Fig. 3:**
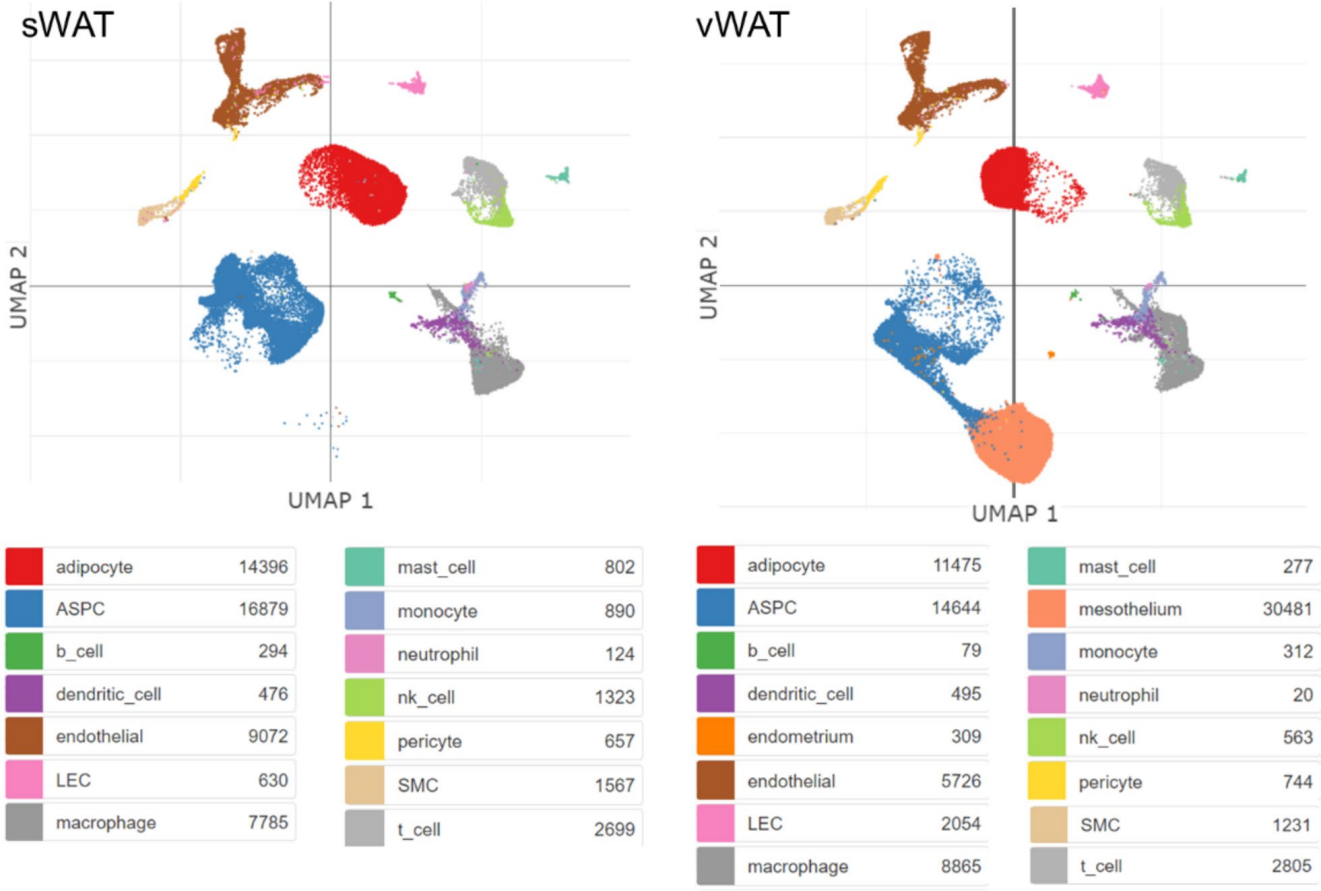
A single cell atlas of adipose tissue. Uniform manifold approximation and projection maps (UMAP) embedding coordinates for human subcutaneous and visceral white adipose tissue (sWAT and vWAT). Image credit: courtesy of Single Cell Portal, www.singlecell.broadinstitute.org/single_cell [138]. The study is available at the following URL: https://singlecell.broadinstitute.org/single_cell/study/SCP1376, accessed: 05 September 2024 [94]

Emont et al. generated a comprehensive atlas of human subcutaneous and visceral WAT on the basis of scRNA and snRNA-seq data [94]. All the main cell populations were represented, to a different extent, in both sWAT and vWAT, excluding mesothelial and endometrial cell clusters present only in visceral fat (Fig. 3). In particular, human white adipocytes cluster into six subpopulations, whereas adipose stem and progenitor cells cluster into seven subpopulations that express the common marker gene platelet-derived growth factor receptor alpha (PDGFRA). Interestingly, no significant difference was observed among the overall proportions of adipocytes and adipose stem and progenitor cells between vWAT and sWAT. Based on this study [94], a reference dataset for the analysis and interpretation of single-cell RNA-seq experiments has been developed and is available on Azimuth (https://app.azimuth.hubmapconsortium.org/app/human-adipose, accessed: 05 September 2024) [95].

More recently, Massier et al*.* generated a high-resolution map of human subcutaneous, omental, and perivascular WAT by analyzing publicly available and newly generated scRNA-seq, snRNA-seq, and spatial transcriptomic data integrated with deconvoluted bulk transcriptomic datasets [96]. This study, which included over 400,000 quality-filtered cells/nuclei obtained from 83 donors, identified more than 60 cell subpopulations, providing a comprehensive overview of the cells residing in human WAT. Remarkably, most of the cell subpopulations were present in all analyzed WAT depots (subcutaneous, omental, and perivascular), as previously observed [94]. Stromal cells, including fibroblast and adipogenic progenitors at different stages of commitment, constituted approximately 40% of the total cell populations, adipocytes were ~ 20%, immune cells ~ 20% and vascular cells less than 15%. Recently, a reference atlas of white adipose tissue including 43 cell subtypes was generated by integrating scRNA-seq and snRNA-seq datasets from 6 independent studies, further supported by deconvoluted bulk RNA-seq data [97]. This study established that, compared with vWAT, sWAT is enriched in vascular endothelial cells, fibro-adipogenic progenitors and T cells.

Single-nucleus RNA sequencing has also been instrumental in defining cell-type specific composition of tumor-adjacent and distal adipose tissue in patients with breast cancer, highlighting the increased expression of inflammatory genes in tumor-associated adipocytes and macrophages [98].

### Adipose tissue spatial transcriptomics

The behavior of adipose tissue mesenchymal stromal cells depends on the local tissue environment, or “niche”, that is characterized by a unique combination of local gradients of signaling factors, nutrients and oxygen, neighboring cell cross-talk, and ECM-derived biomechanical forces. Single-cell omics studies contribute to the accurate definition of gene expression within each cell but cannot capture the cell location information that is lost during tissue disaggregation required for sample processing. Spatial transcriptomics combines RNA sequencing and immunohistological analysis of intact tissue sections, mapping transcriptional data to their original spatial location within the tissue, ideally at single-cell resolution. Thus, spatial transcriptomics allows the investigation of tissue spatial heterogeneity and provides insights into cell–cell interactions and tissue functional organization.

Three distinct adipocyte and two adipocyte progenitor subpopulations were identified in human WAT; interestingly, spatial transcriptomics analysis confirmed that collagen-rich adipocyte progenitors are frequently positioned over fibrous and vascular structures and close to M2-like macrophages [99], organizing adipogenic niches [100]. More recently, Massier et al*.* described an integrated single cell and spatial transcriptomic map of human subcutaneous, omental, and perivascular WAT [96]. In particular, the authors identified in scWAT 17 clusters of fibroblasts and adipogenic stem/progenitor cells at different stages of commitment (FAPs) with specific tissue distributions, commonly near to vessels or macrophages, forming distinct putative regenerative niches.

## Adipose tissue proteomics and secretomics

Transcriptomic data cannot precisely predict cellular protein content because multiple regulatory processes in transcription, translation, post-translational modification, mRNA and protein degradation affect protein production and stability. Characterization of adipose tissue, ASCs and their secretome using high-throughput mass spectrometry-based proteomics has been performed, as previously reviewed [32, 101]. Some studies have analyzed fully digested protein preparations from adipose tissue and have demonstrated that the levels of relevant metabolic pathways, including carbohydrate and lipid metabolism, protein synthesis, and inflammation, are increased in VAT compared with SAT, in agreement with an higher cardiovascular risk associated with visceral adiposity [102, 103]. Other studies have investigated the proteome in isolated cellular fractions [104] and have shown that mature adipocytes and SVF cells share a common proteomic profile [105]. In particular, adipokines are produced both by adipocytes and SVF cells, whereas inflammatory mediators, such as tumor necrosis factor-α (TNF-α) and interleukin-6 (IL-6), are mainly produced by SVF cells [106]. Moreover, proteomic analysis highlighted significant differences between uncultured adipocytes isolated from different obese donor-matched adipose tissue depots; in particular, the vWAT adipocyte proteome has an increased content of proteins involved in glycolysis, lipogenesis, and mitochondrial function compared with sWAT [107]. Approximately 22% of the total number of proteins in adipocytes have mitochondrial origin, compared with 4.8% in the whole human proteome [108], confirming the important role of mitochondria in key pathways such as lipid and glucose homeostasis and oxidative capacity [109]. More recently, comparison between sWAT and vWAT proteomes from lean and obese individuals and patients with type 2 diabetes revealed specific down-regulation of mitochondrial and up-regulation of ECM and immune response-related pathways associated with type 2 diabetes [110].

Adaptation to cell culture involves proteome changes, as assessed during amplification under good manufacturing practices of ASCs that otherwise maintain a homogeneous immunophenotypic profile up to seven passages [111]. Dynamic proteomic changes in ASCs undergoing adipocyte differentiation indicate modulation of Wnt and transforming growth factor-β (TGF-β) signaling pathways [112], whereas osteogenic differentiation is associated with increased expression of integrins and genes involved in ECM organization [113].

Adipose tissue releases hundreds of bioactive molecules, commonly referred to as adipokines, that contribute to the regulation of whole-body energy metabolism and mediate ASCs regenerative and immunomodulatory potential. The adipose tissue secretome comprises a wide array of proteins, peptides, metabolites, lipids (lipokines), and extracellular vesicle cargoes, including non-coding RNAs [114, 115].

The use of ASC conditioned medium, EVs and decellularized lipoaspirate has gained attention as a potential cell-free alternative to cell therapy [116, 117]; before clinical translation, precise assessment of adipose tissue/ASC secretome is mandatory and standardization is required [118]. Transcript levels do not correlate with secreted protein levels because proteins may be stored in secretory vesicles and released upon stimulation; therefore, adipose tissue transcriptomics should be integrated with proteomic studies of secreted proteins (*secretomics*). The secretome collected from in vitro cell cultures only partially reflects the in vivo complexity, whereas the adipose tissue secretome contains proteins released by both adipocytes and SVF cells. Adipokines released by vWAT explants include adiponectin, interleukin-6, and proteins involved in ECM organization [119]. Proteomic evaluation of extracellular vesicles produced in vitro by human primary adipocytes identified over 880 proteins, among which fatty acid binding protein 4 (FABP4) has been proposed as a tissue-specific marker [120].

Cellular protein content is highly dynamic; although the average protein copy number per cell is higher than the corresponding number of RNA copies, proteins cannot be amplified like nucleic acids, making the assessment of the proteome in individual cells difficult. Nonetheless, recent technical developments have rapidly moved the field toward single-cell proteomics through liquid chromatography mass spectrometry and next-generation sequencing, single-cell spatial proteomics [121], and time-resolved assessment of protein secretion from single cells by sequencing (TRAPS-seq) [122]. Therefore, single-cell-based proteomic studies of human adipose tissue will soon contribute in deciphering adipose tissue heterogeneity.

## Adipose tissue lipidomics

Adipocytes exhibit highly dynamic lipid metabolism that involves the production, storage, and release of bioactive lipids (*lipokines*) that modulate immunological function and tissue homeostasis. In 2021, Lange et al. [123] presented a mass spectrometry-based atlas of the lipidome of human adipose tissue that captures the lipid diversity of over 1600 lipid molecular species, mainly triacylglycerols, whose physiological contributions remain largely unexplored. Lipid composition is depot-specific and heterogeneous [124]; for instance, differences in plasmalogen content in sWAT and vWAT affect cell membrane fluidity and, in turn, adipocyte remodeling and pathological fat mass expansion [123]. Despite the progress of mass spectrometry methods, the identification of lipids at the single-cell level remains difficult and, to the best of our knowledge, analysis of human adipose tissue has not been performed yet [125].

## Adipose tissue matrisomics

Adipose tissue extracellular matrix (ECM) is a complex meshwork mainly composed of collagens, fibronectin, laminin and glycosaminoglycans. ECM organizes tissue 3D architecture, supports tissue growth and remodeling in response to nutritional status, and provides biomechanical, biochemical, and biophysical cues to coordinate cell proliferation, migration and differentiation during tissue repair. Decellularized adipose tissue/ASC ECM-derived products have reduced immunogenicity and may be employed for the fabrication of biocompatible scaffolds for regenerative medicine using innovative three-dimensional bioprinting techniques [126, 127]. To obtain a decellularized matrix, cellular, genetic, and lipidic components of adipose tissue are removed by enzymatic, physical, or chemical procedures, while the native 3D configuration of the ECM is preserved [128]. No consensus has been reached regarding the best procedure for obtaining an adipose tissue-derived material that preserves the native microenvironment to support in situ tissue regeneration. Standardization of the procedures and characterization of the adipose tissue matrisome are essential to develop a decellularized ECM suitable for translational procedures [129]. In particular, significant matrisome differences were detected between suction and power-assisted lipoaspirates. The most abundant proteins in the adipose tissue matrisome are structural collagens and heparan sulphate proteoglycan, whereas the content of some proteins, such as fibronectin and vitronectin, varies according to donor age and depot location [129].

Clinical use of off-the-shelf decellularized adipose-derived products in regenerative medicine procedures is ongoing; precise definition of the ECM components, their biomechanical proprieties, involvement in tissue repair, degradation time, immune profile, and standardization of product development will further promote clinical translation [130].

## Perspectives and conclusions

In recent years, omics-based approaches at single-cell and spatial resolutions have been applied to dissect human adipose tissue heterogeneity and provide insights into ASC differentiation trajectories [11]. Technologies are being further refined to increase the throughput requirements needed to characterize rare subpopulations, reduce costs, and minimize sample bias. Currently, several methods are under investigation to reduce batch effects by integrating scRNA-seq and snRNA-seq datasets acquired with different technologies and annotations [97]. Moreover, computational advances, artificial intelligence integration, and improved spatial transcriptome deconvolution algorithms will allow the extension of data analysis of spatial single-cell heterogeneity. For instance, spatial transcriptomic and proteomic data across several tissue slices can be analyzed utilizing deep learning approaches to generate multiomic 3D maps. The next frontier is currently represented by the integration of simultaneous multimodal spatial single-cell omics approaches [9] and the development of technologies with improved resolution for subcellular omics [131] to elucidate the key role of mitochondria in human WAT function [109]. Combined simultaneous application of different omics technologies on human adipose tissue is increasing. Of particular relevance, are studies that associate transcriptome/genome and methylome analyses to identify possible epigenetic differences in different adipose tissue depots under pathological condition [69, 132].

However, several critical points need to be further addressed: in general, single-cell analysis describes a snapshot of the cellular gene expression profile, whereas the adipose tissue cellular landscape is highly dynamic in response to different stimuli. Therefore, adipose tissue heterogenicity may arise, at least in part, by cell transition into different functional states, which require extensive studies to be fully captured. In addition, the tissue microenvironment clearly plays a critical role in regulating the ASC state; therefore, 3D information should be preserved for understanding possible cell–cell interactions in the context of tissue structural organization.

Notwithstanding these limitations, single-cell omics studies have provided formal evidence of the extensive cellular heterogeneity of adipose tissue (Table 3) encompassing over 60 different subpopulations of adipocytes, fibroblast (stromal) and adipogenic progenitors, vascular, and immune cells [96]. Single-nucleus RNA sequencing studies confirmed observations from morphological studies, indicating that, despite occupying most of the tissue volume, adipocytes represent only approximately 20% of the total WAT cell number. Different mature adipocyte subclusters have been identified in each depot, possibly associated with heterogeneous functional differences [133]. Conversely, according to both snRNA-seq and scRNA-seq data, fibroblast and adipogenic precursors numerically represent the largest class of WAT cell populations. Overall, all cell subpopulations were present in both sWAT and vWAT, but some depot-specific differences were observed. In particular, vWAT is enriched with mesothelial cells and macrophages, whereas sWAT is enriched with vascular, fibro-adipogenic progenitors, and endothelial cells [97], suggesting that WAT is a preferable source of progenitor cells for regenerative applications. Moreover, sWAT spatial transcriptomic data indicate that fibro-adipogenic progenitors are located in proximity to vessels, in accordance with the previously established association of mesenchymal cells with perivascular niches [134].

In conclusion, recent studies using single‐cell multiomic approaches have significantly advanced our understanding of the cellular heterogeneity within the human white adipose tissue stromal vascular fraction. Even if the assessment of the clinical relevance of the identified cell types needs to be fully established, these studies may lead to the potential selection of unique subpopulations with optimized regenerative potentials, paving the way to more reproducible and effective clinical applications using adipose tissue derivatives.

## Data Availability

Not applicable.

## Abbreviations

ASC: Adipose tissue-derived stromal cell
ECM: Extracellular matrix
EV: Extracellular vesicle
FACS: Fluorescence-activated cell sorting
MSC: Mesenchymal stromal cell
PPAR-γ: Peroxisome proliferator-activated receptor-gamma
scRNA-seq: Single-cell RNA sequencing
snRNA-seq: Single-nucleus RNA sequencing
SAT or sWAT: Subcutaneous (white) adipose tissue
SVF: Stromal vascular fraction
VAT or vWAT: Visceral (white) adipose tissue
WAT: White adipose tissue
